# Estrogen Receptor-β Gene Cytosine-Adenine (*ESR2*-CA) Repeat Polymorphism in Postmenopausal Colon Cancer

**DOI:** 10.3390/ijms24054502

**Published:** 2023-02-24

**Authors:** Naoko Honma, Tomio Arai, Yoko Matsuda, Yosuke Fukunaga, Masaaki Muramatsu, Shinobu Ikeda, Yuri Akishima-Fukasawa, Noriko Yamamoto, Hiroshi Kawachi, Yuichi Ishikawa, Kengo Takeuchi, Tetuo Mikami

**Affiliations:** 1 Department of Pathology, Faculty of Medicine, Toho University, Omori-Nishi, Ota-ku, Tokyo 143-8540, Japan; 2 Division of Pathology, Cancer Institute, Japanese Foundation for Cancer Research, Ariake, Koto-ku, Tokyo 135-8550, Japan; 3 Department of Pathology, Tokyo Metropolitan Geriatric Hospital, Sakaecho, Itabashi-ku, Tokyo 173-0015, Japan; 4 Oncology Pathology, Department of Pathology and Host-Defense, Faculty of Medicine, Kagawa University, Ikenobe, Miki-cho, Kita-gun, Kagawa 761-0793, Japan; 5 Gastroenterological Center, Department of Gastroenterological Surgery, Cancer Institute Hospital, Japanese Foundation for Cancer Research, Ariake, Koto-ku, Tokyo 135-8550, Japan; 6 Diagnostics and Therapeutics of Intractable Diseases, Intractable Disease Research Center, Graduate School of Medicine, Juntendo University, Hongo, Bunkyo-ku, Tokyo 113-8421, Japan; 7 Japan Agency for Medical Research and Development, Otemachi, Chiyoda-ku, Tokyo 100-0004, Japan; 8 Department of Pathology, Cancer Institute Hospital, Japanese Foundation for Cancer Research, Ariake, Koto-ku, Tokyo 135-8550, Japan; 9 Department of Pathology, International University of Health and Welfare, Mita, Minato-ku, Tokyo 103-8329, Japan; 10 Pathology Project for Molecular Targets, Cancer Institute, Japanese Foundation for Cancer Research, Ariake, Koto-ku, Tokyo 135-8550, Japan

**Keywords:** age, colon cancer, estrogen, estrogen receptor-β, *ESR2*-CA repeat polymorphism, postmenopausal women, mismatch repair protein

## Abstract

The pathobiological role of estrogen is controversial in colorectal cancer. Cytosine-adenine (CA) repeat in the estrogen receptor (ER)-β gene (*ESR2*-CA) is a microsatellite, as well as representative of *ESR2* polymorphism. Though its function is unknown, we previously showed that a shorter allele (germline) increased the risk of colon cancer in older women, whereas it decreased it in younger postmenopausal women. *ESR2*-CA and ER-β expressions were examined in cancerous (Ca) and non-cancerous (NonCa) tissue pairs from 114 postmenopausal women, and comparisons were made considering tissue types, age/locus, and the mismatch repair protein (MMR) status. *ESR2*-CA repeats <22/≥22 were designated as ‘S’/‘L’, respectively, resulting in genotypes SS/nSS (=SL&LL). In NonCa, the rate of the SS genotype and ER-β expression level were significantly higher in right-sided cases of women ≥70 (≥70Rt) than in those in the others. A decreased ER-β expression in Ca compared with NonCa was observed in proficient-MMR, but not in deficient-MMR. In NonCa, but not in Ca, ER-β expression was significantly higher in SS than in nSS. ≥70Rt cases were characterized by NonCa with a high rate of SS genotype or high ER-β expression. The germline *ESR2*-CA genotype and resulting ER-β expression were considered to affect the clinical characteristics (age/locus/MMR status) of colon cancer, supporting our previous findings.

## 1. Introduction

Estrogens have attracted attention as factors affecting the risk and outcome of colorectal cancer (CRC) [1,2,3,4,5,6]. A large number of in vitro and in vivo studies have suggested the inhibitory role of estrogens against CRC [1,2,4,7,8], whereas a fewer, but significant, number of studies have reported unfavorable effects on CRC [6]. CRC characteristics are affected by sex, age, and tumor locus. The proportion of right-sided colon cancer increases as age increases, and is consistently higher in women than men regardless of age [6]. Histologically, the proportion of medullary/mucinous carcinoma (Med/Muc), which frequently locate right-sided, increases with age. Microsatellite instability (MSI), a representative carcinogenic mechanism caused by deficiency of mismatch repair protein (dMMR), is associated with CRC of women, right-sidedness, or Med/Muc histology. Further, the concentration of estrogens drastically fluctuates throughout a woman’s lifetime: much higher in premenopausal women, but much lower in postmenopausal women than men. In such context, a systematic study considering sex, age, tumor locus, or MMR status is needed to appropriately understand the pathobiological role of estrogens in CRC.

In normal colorectal epithelium, estrogen receptor (ER)-β, the second ER, is the main ER, suggesting that estrogen exerts its function through ER-β. ER-β expression has been reported to decrease according to canceration or cancer progression, suggesting that the estrogen-ER-β signaling malfunction is related to the carcinogenesis/development of CRC [1]. Further, expression of ER-β1, a wild type ER-β, has been reportedly higher in MSI-positive than MSI-negative CRC [1,9]. In an epidemiological study, estrogen has been suggested to reduce MSI-negative or ER-β-positive CRC, but not MSI-positive or ER-β-negative CRC [10,11]. These findings suggest that estrogen affects the tumor types generated, as well as behaves differently according to the status of MSI or ER-β.

We recently examined the estrogen concentration and expression of the estrogen receptor (ER)-β (the main ER in colorectum) in pairs of colon cancerous/non-cancerous tissues (Ca/NonCa) from postmenopausal women. In such a study setting, we could avoid the effect of gender difference or menopausal status, and could focus on the effect of age, tumor locus, or tumor type. ER-β reduction in Ca compared with its NonCa counterpart, which has been repeatedly reported, was observed only in left-sided cases involving patients younger than 70 y/o, cases with a non-medullary/mucinous (Med/Muc) histology, or MMR-proficient (pMMR) cases, suggesting that the inhibitory role of estrogens is limited to these types of tumors. By contrast, ER-β-positivity, higher estradiol (E2) concentration, dMMR, and Med/Muc histology were closely related to right-sided tumors in women 70 y/o or older (≥70Rt), and those factors were also closely related to each other, suggesting a promotive role of estrogens in these tumors [12]. Groups including ours previously reported that ER-β gene (*ESR2*) cytosine-adenine (CA) repeat polymorphism (*ESR2*-CA) [13] in the germline affected the colon cancer risk in postmenopausal women, but not rectal cancer risk, or colon cancer risk in men and premenopausal women [14,15,16]. Further, the risk of this polymorphism in postmenopausal colon cancer has been shown to invert with age: a shorter allele (S) was associated with a higher risk in older women, but a lower risk in younger postmenopausal women [14,15,16], suggesting a divergent pathogenic role of this polymorphism in postmenopausal colon cancer according to age.

*ESR2*-CA is one of the microsatellites. Microsatellites instability (MSI), a change in the repetition number of microsatellites in tumors, is caused by a deficiency of MMR. Lynch syndrome, or hereditary non-polyposis colorectal cancer (HNPCC), is well-known for its germline mutation; however, MMR-deficiency is also frequently noted in non-hereditary colon cancer in older women, with a right-sided locus, or with Med/Muc histology. Although the germline *ESR2*-CA and risk of colon cancer have been associated, as described above, the instability of this microsatellite in colon cancerous tissue has never been reported. The purpose of this study was to elucidate whether and how *ESR2*-CA or its instability affects colon cancer pathogenesis, considering the background of patients and tumors. We also examined the association between *ESR2*-CA and ER-β expression, which has also never been reported.

## 2. Results

### 2.1. Patient/Tumor Background

The association between the MMR status and patient/tumor background considering age and tumor locus is summarized in Table 1. The rate of dMMR was significantly different among four groups (≥70Rt, <70/right (<70Rt), ≥70/left (≥70Lt), and <70/left (<70Lt). *p* = 0.0023), and higher in ≥70Rt than in the other three groups, suggesting the specificity of ≥70Rt. These groups other than ≥70Rt were combined as Non(≥70Rt). The rate of dMMR was again significantly higher in ≥70Rt than in Non(≥70Rt) (*p* = 0.0005). Additionally, taking tissue categories (Ca and NonCa) and the MMR status (dMMR and pMMR) into consideration, we further categorized samples into eight groups, and comparisons were made among these groups in the following analyses.

### 2.2. ESR2-CA

#### 2.2.1. Distribution of ESR2-CA Allele Frequency

**Figure 1:**
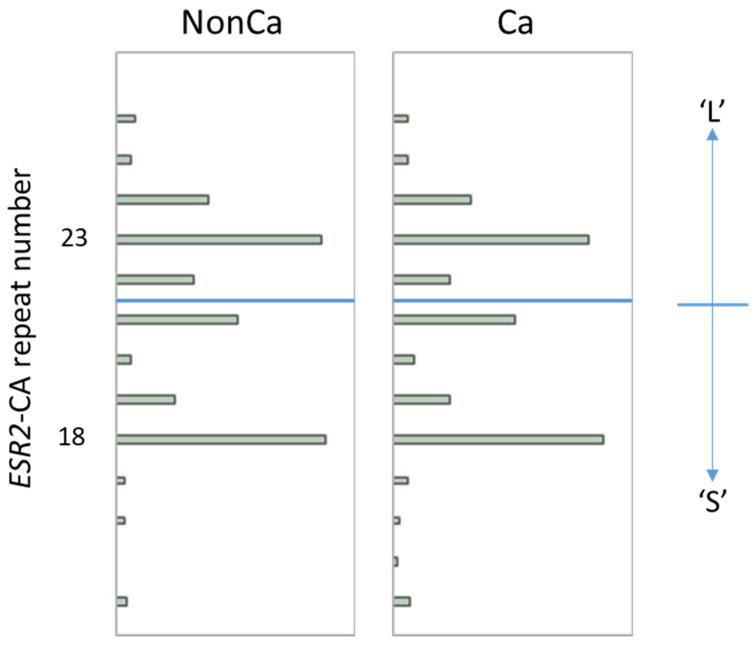
The distribution of *ESR2* cytosine-adenine (*ESR2*-CA) allele frequency in non-cancerous (NonCa) and cancerous (Ca) tissue, with boxplots. Two major peaks were observed at 18 and 23 in both tissue categories, which is largely consistent with the results of other studies in the Japanese population. Alleles with CA repeats <22 and ≥22 were designated as ‘S’ and ‘L’, respectively, resulting in genotypes SS, LS, and LL. Genotypes ‘SL’ and ‘LL’ were combined as ‘nSS’.

The distribution of the *ESR2*-CA allele frequency in NonCa and Ca is shown in Figure 1. In both tissue categories, the number of ESR2 CA repeats was distributed from 14 to 26 with two major peaks at 18 and 23, which is largely consistent with the results of other studies in the Japanese population (Figure 1) [14,15,17]. Using the same cutoff as in previous Japanese studies [14,15,17], we designated the allele with CA repeats <22 as the ‘S’ allele and ≥22 as the ‘L’ allele (Figure 1), resulting in three genotypes, SS, SL, and LL. To simplify this, SL and LL were combined as ‘nSS’, and the subjects were divided into two genotype categories, ‘SS’ and ‘nSS’.

#### 2.2.2. Change of ESR2-CA Repeat Number in Ca Compared with NonCa

**Figure 2:**
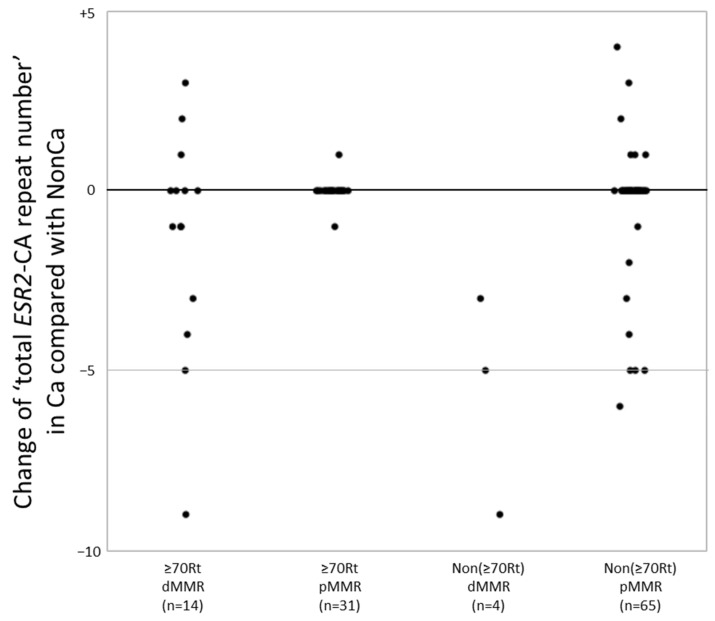
The ‘total *ESR2*-CA repeat number’ in cancerous tissue (Ca) was compared with that in non-cancerous tissue (NonCa) in each patient. The distribution of the difference of ‘total *ESR2*-CA repeat number’ between Ca and NonCa was compared among four groups categorized by the age/locus and mismatch repair protein (MMR) status. dMMR, deficient-MMR; pMMR, proficient-MMR; ≥70Rt, right-sided case ≥ 70 y/o; Non(≥70Rt), other than ≥70Rt.

A change in the number of CA repeats between NonCa and Ca was significantly more frequent in dMMR cases (14/18, 78%) than in pMMR cases (16/96, 17%) (*p* < 0.0001). In more detail, 10 of 14 ≥70Rt/dMMR (71%), 2 of 31 ≥70Rt/pMMR (6.4%), four of four Non(≥70Rt)/dMMR (100%), and 14 of 65 Non(≥70Rt)/pMMR (22%) exhibited change in the number of CA repeats between NonCa and Ca, yielding a significant difference among the four categories (*p* < 0.0001). To examine more precisely, we added the number of two *ESR2*-CA repeats in each sample to yield ‘total *ESR2*-CA repeat number’, and compared it between the pairs (NonCa and Ca) of each patient. The distribution of the difference of ‘total *ESR2*-CA repeat number’ between pairs was compared among the four categories (Figure 2). In ≥70Rt/dMMR, 10 of 14 exhibited −9 to +3, whereas only one each of ≥70Rt/pMMR showed +1 and −1. All 4 Non(≥70Rt)/dMMR exhibited a decrease in CA repeat number in tumors (−9 to −3), whereas 14 of 65 Non(≥70Rt)/pMMR showed various differences between pairs.

#### 2.2.3. Comparison of ESR2-CA Genotype among Categories

**Figure 3:**
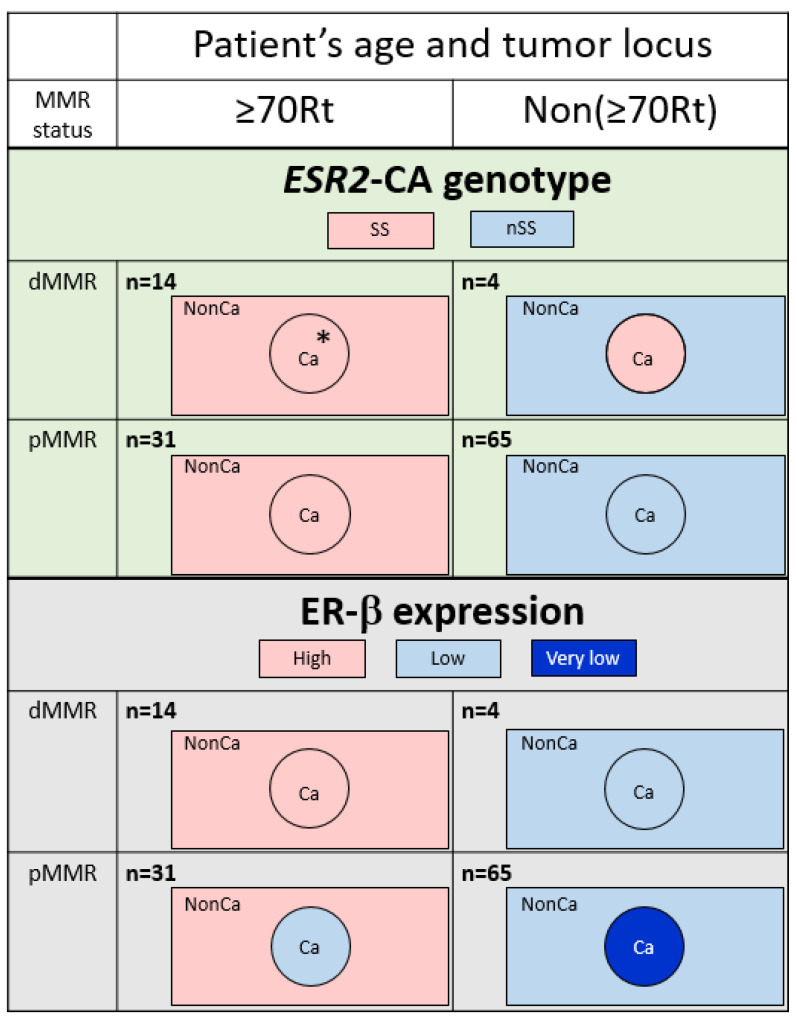
Typical feature of *ESR2* cytosine-adenine (*ESR2*-CA) genotype (**above**) and ER-β expression (**below**) according to the categories determined by patients’ age/tumor locus (right-sided case ≥70 y/o, ≥70Rt vs. the others, Non(≥70Rt)) and mismatch repair protein status (MMR-deficient, dMMR vs. MMR-proficient, pMMR). The number of *ESR2*-CA repeats frequently varied in dMMR tumors; however, no change was produced in the genotype in ≥70Rt/dMMR (*). A decrease of ER-β expression in Ca compared with NonCa was observed only in pMMR tumors. Ca, cancerous tissue; NonCa, non-cancerous tissue.

The *ESR2*-CA genotype was compared among four groups categorized by the age/locus and MMR status in NonCa and Ca (Table 2). In NonCa, the proportion of SS was larger in ≥70Rt than in Non(≥70Rt) (*p* = 0.0007) irrespective of the MMR status. In Ca, the proportion of SS was also higher in ≥70Rt than in Non(≥70Rt); however, the difference was much less in Ca than in NonCa (*p* = 0.0515) because of cases with changes of the *ESR2*-CA genotype in Non(≥70Rt) tumors (nSS in NonCa to SS in Ca). The *ESR2*-CA genotype was also compared between Ca and NonCa among the same group categorized by age/locus and the MMR status. The proportion of SS was same between NonCa and Ca in ≥70Rt (*p* = 1.0000), but was insignificantly higher in Ca than NonCa among Non(≥70Rt) (*p* = 0.1096) (Table 2, Figure 3, above).

### 2.3. Expression of ER-β

**Figure 4:**
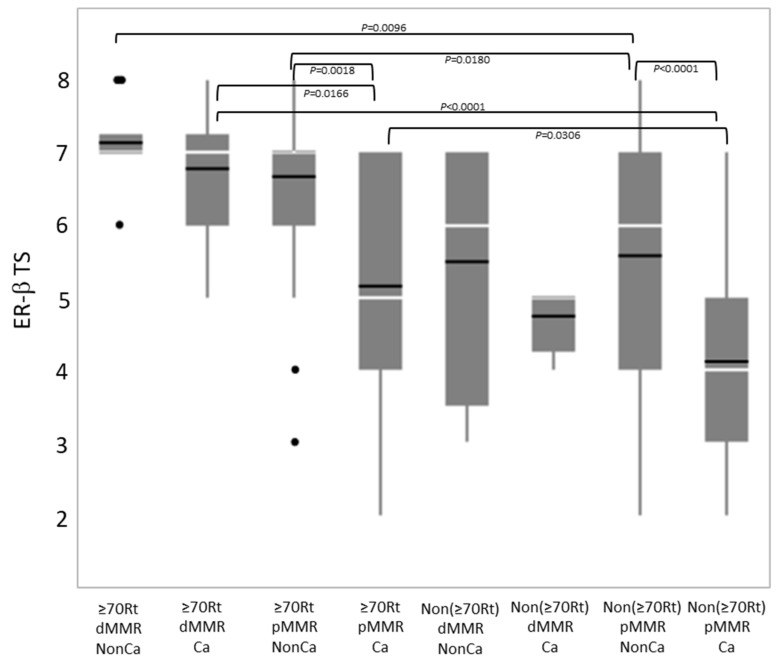
Comparison of immunohistochemical ER-β expression (total score, TS) by boxplot among eight groups categorized by tissue type (Ca vs. NonCa), age/locus (≥70Rt vs. Non(≥70Rt)), and MMR status (dMMR vs. pMMR). White horizontal bar, median; black horizontal bar, medium. Ca, cancerous tissue; NonCa, non-cancerous tissue; ≥70Rt, right-sided case ≥ 70 y/o; Non(≥70Rt), other than ≥70Rt; dMMR, mismatch repair protein (MMR)-deficient; pMMR, MMR-proficient.

Immunohistochemical ER-β expression (total score) was compared among eight groups categorized by tissue type, age/locus, and the MMR status (Figure 4). Among NonCa, ≥70Rt/dMMR and ≥70Rt/pMMR exhibited significantly higher ER-β expression than Non(≥70Rt)/pMMR. Among Ca, ≥70Rt/dMMR and ≥70Rt/pMMR again exhibited significantly higher ER-β expression than Non(≥70Rt)/pMMR, and furthermore, ER-β expression in ≥70Rt/dMMR was significantly higher than in ≥70Rt/pMMR. As for the comparison between NonCa and Ca, a significant decrease in Ca was observed in ≥70Rt/pMMR and Non(≥70Rt)/pMMR, but not in ≥70R/dMMR or Non(≥70Rt)/dMMR (Figure 3 (below) and Figure 4).

### 2.4. The Relation between ESR2-CA Genotype and ER-β Expression

**Figure 5:**
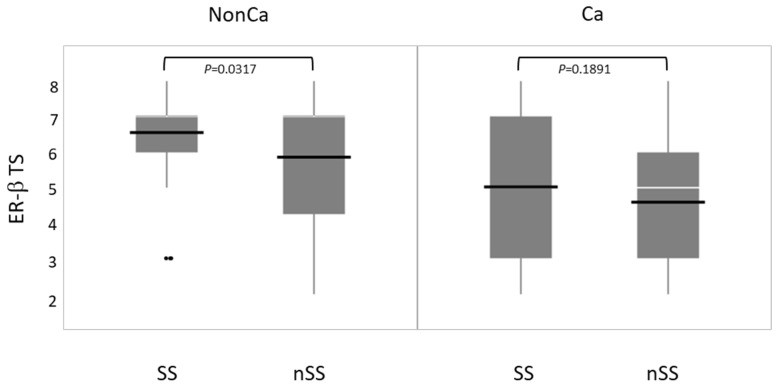
The relation between *ESR2* cytosine-adenine (*ESR2*-CA) genotype and ER-β expression (total score, TS) in non-cancerous (NonCa) and cancerous (Ca) tissues by boxplots. White horizontal bar, median; black horizontal bar, medium. In NonCa, but not in Ca, ER-β expression was significantly higher in SS than in nSS. Ca, cancerous tissue; NonCa, non-cancerous tissue.

Immunohistochemical ER-β expression (total score, TS) was compared between SS and nSS in NonCa and Ca. ER-β expression was significantly higher in SS than in nSS among NonCa, which was not true among Ca (Figure 5).

## 3. Discussion

In the present study, we examined the *ESR2*-CA and ER-β expression in NonCa and Ca of surgical materials from postmenopausal colon cancer patients, taking the patients’ age, tumor locus, and MMR status into consideration. This is the first study to systematically compare *ESR2*-CA, one of the microsatellites, between NonCa and Ca, and to compare it with ER-β expression. We observed that: (1) in NonCa, the rate of the *ESR2*-CA SS genotype and ER-β expression level were significantly higher in ≥70Rt than in Non(≥70Rt) irrespective of the MMR status (Table 2, Figure 3 and Figure 4). (2) In NonCa, ER-β expression was significantly higher in SS than in nSS, which was not true in Ca (Figure 5). (3) The *ESR2*-CA repeat number frequently differed between NonCa and Ca in dMMR, but not in pMMR, although the *ESR2*-CA genotype did not significantly differ between them irrespective of MMR status (Table 2, Figure 2 and Figure 3).

We previously showed that germline SS increased the risk of colon cancer in older women as opposed to younger postmenopausal women, in whom SS decreased the risk [14,15]. We also showed that most colon cancers in older women with the germline SS genotype were right-sided, and exhibited higher ER-β expression in Ca, as well as in NonCa, although the sample size was small [14]. These previous findings are consistent with the present results, whereby the frequency of the SS genotype and ER-β expression levels were high in ≥70Rt than Non(≥70Rt) irrespective of the tissue category (Table 2, Figure 3 and Figure 4). In NonCa, ER-β expression was significantly higher in SS than in nonSS (Figure 5), suggesting that *ESR2*-CA determines ER-β expression, which leads to estrogen activity, at least partly, in normal colon epithelium. An association between shorter *ESR2*-CA alleles and higher estrogen activity has been suggested by studies on bone mineral density or systemic lupus erythematosus (SLE) [18,19]. The biological role of *ESR2*-CA, which exists in intron 5 of the *ESR2* locus, is not known at present; however, intronic microsatellite repeats have been suggested to alter gene transcription, mRNA splicing or translation, gene silencing, or interaction with coregulators [20]. Several isotypes are known for ER-β. Ligand binding domain (LBD) is coded by alternatively spliced exon 8 of ESR2, resulting in five different forms of ER-β: ER-β1 to ER-β5 [21,22]. ER-β1, the wild-type, can bind to estrogens and transduces their signals; however, ERβ2-5 variants, with a truncated form of this domain, lack binding ability and dominant negatively regulate estrogen signaling [23]. One of the attractive hypotheses is that, in NonCa, *ESR2*-CA SS (but not nSS) facilitates the alternative splicing of exon 8, increasing the expression of wild-type ER-β (ER-β1), which is recognized by clone PPG5/10 used in this study as the primary antibody against ER-β [18,24].

By contrast, in Ca, ER-β expression was not related to *ESR2*-CA, suggesting a disordered relation between them (Figure 5). In Ca with pMMR, despite unchanged *ESR2*-CA, ER-β expression was significantly lower than in NonCa irrespective of patients’ age, which was not true in Ca with dMMR where ER-β expression was stable. The finding that the *ESR2*-CA repeat number frequently changed in Ca with dMMR but not in Ca with pMMR is reasonable because *ESR2*-CA is one of the microsatellites, which are used as indicators of the MMR status (MSI). The change in the *ESR2*-CA repeat number, however, did not affect the *ESR2*-CA genotype in ≥70Rt/dMMR. Although the mechanisms of how ER-β expression is regulated in Ca is unclear, a decrease in the estrogen action may be pathogenically important in pMMR tumors, but not in dMMR tumors. In pMMR, a carcinogenic mechanism other than MMR might cause an abnormality which disturbs the normal transcription of *ESR2*.

**Figure 6:**
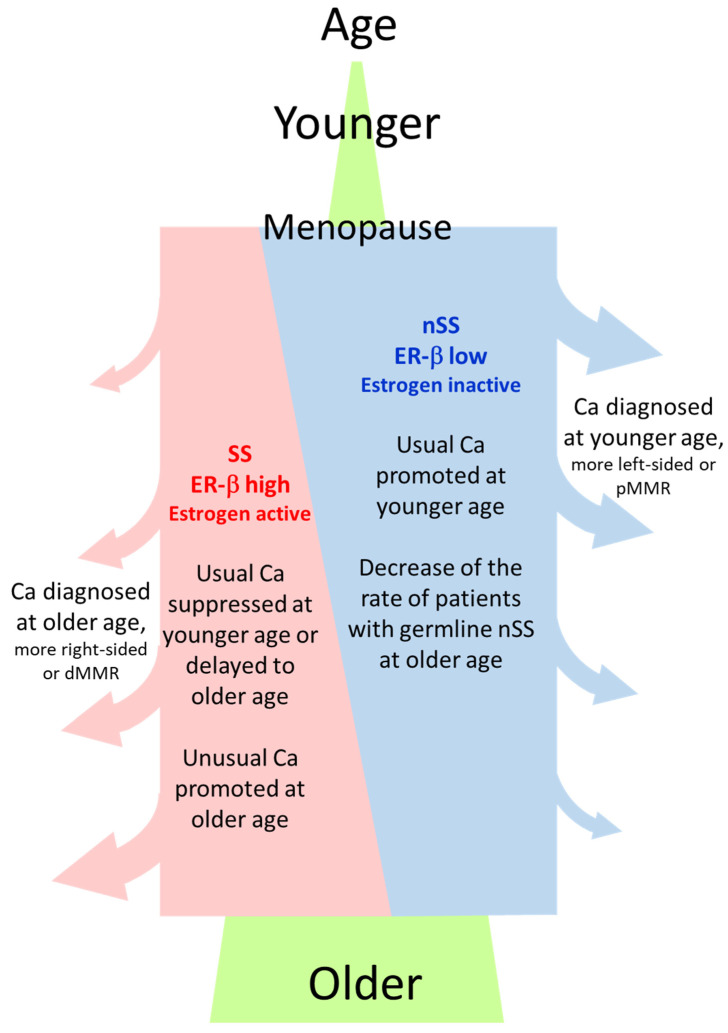
General image of the relation between *ESR2*-CA genotype (SS or nSS) in NonCa, age at cancer diagnosis, and cancer type. ‘Usual Ca’, having characteristics such as left-sided or pMMR, is suppressed by estrogen, whereas ‘unusual Ca’, having characteristics such as right-sided or dMMR, is promoted by estrogen. Postmenopausal women with nSS genotype and lower ER-β expression in NonCa have a higher risk of usual Ca at a younger age, contrasting with those with the SS genotype, where more Ca are diagnosed at an older age due to slow onset/development of the disease. Ca, cancerous tissue; NonCa, non-cancerous tissue; dMMR, mismatch repair protein (MMR)-deficient; pMMR, MMR-proficient.

Why was the rate of the *ESR2*-CA SS genotype or ER-β expression high in NonCa in ≥70Rt? One hypothesis is that the germline SS genotype and resulting higher ER-β expression predisposes NonCa in ≥70Rt to cancerization, which means SS/ER-β promotes carcinogenesis in ≥70Rt. The right-sided colon is generally thought to be affected by the environment more than the left-sided colon or rectum. NonCa in ≥70Rt is likely to be most affected by the environment because of longtime exposure according to age. We previously showed that the estrogen concentration was high in Ca in ≥70Rt. Longtime exposure to a high estrogen concentration, which also contributes to increased nuclear ER-β expression, may be pathogenically important for cancer in ≥70Rt. In an experimental study using a colon cancer cell line with low ER-β expression, compulsory ER-β expression decreased MMR [25]. The dMMR tumor in ≥70Rt is an attractive candidate to explain this hypothesis because it has the background of NonCa with the *ESR2*-CA SS genotype, high ER-β expression, and a high estrogen concentration. Another hypothesis is that SS/ER-β lowers the risk of ‘usual’ cancer at a younger age by delaying the onset or suppressing proliferation until an advanced age when cancer is finally diagnosed. The pMMR tumor in ≥70Rt is a candidate to explain the second hypothesis. Alternatively, both of these hypotheses may work in coordination to develop Ca in ≥70Rt (Figure 6). By contrast, in women with the nSS genotype, low ER-β expression and menopausal estrogen decreases may increase the risk of ‘usual’ type colon cancer soon after menopause, suggesting the protective role of estrogen against colon cancer as generally believed (Figure 6) [1,2,4,7,8].

We speculate that the SS genotype is prone to develop dMMR tumors in ≥70Rt through active estrogen-ER-β signaling, as described above. Estrogen was suggested to promote dMMR through hMLH1 promoter methylation in prostatic/ovarian carcinogenesis [26,27]; however, the mechanism by which estrogen promotes hMLH1 promoter methylation is unclear. The relationship between *ESR2*-CA repeat and methylation status is also unknown. It deserves further study to clarify these associations because dMMR-related CRC in older patients is mostly caused by hMLH1 promoter methylation.

Is a change of the *ESR2*-CA repeat number, one of MSI, important in the pathogenesis of colon cancer? In the present study, this MSI seems to be the result of MMR, and not pathogenically important for the disease, because it neither significantly affected *ESR2*-CA genotype nor related with ER-β expression in tumors.

In the present study, the germline *ESR2*-CA genotype and resulting ER-β expression/estrogen activity was suggested to affect the type of colon cancer generated or patients’ age. Further studies to clarify the precise mechanism are needed to control colon cancer by manipulating estrogen.

## 4. Materials and Methods

### 4.1. Subjects

Pathological materials and frozen samples (pairs of Ca and NonCa) from 114 postmenopausal Japanese women (≥70 y/o, n = 72; <70 and ≥55 y/o, n = 42) who underwent curative surgery for colon cancer without preoperative therapy between 2006 and 2013 were available for this study at the Department of Pathology, Tokyo Metropolitan Geriatric Hospital and Cancer Institute Hospital, Japanese Foundation for Cancer Research (Tokyo, Japan). Histological classification was based on the Japanese Classification of Colorectal, Appendiceal, and Anal Carcinoma [28].

### 4.2. Immunohistochemical Examination of ER-β and MMR

Immunostaining was performed for representative sections of formalin-fixed and paraffin-embedded tissue using an autostainer, BOND III (Leica Microsystems Ltd., Shanghai, China), as described elsewhere [12]. Briefly, an anti-ER-β1 mouse monoclonal antibody was used to detect ER-β (mouse, clone PPG5/10; Bio-Rad Laboratories, Hercules, CA, USA. X20). MMR was detected by monoclonal antibody for MLH1 (rabbit, clone EPR3894; Abcam plc., Cambridge, UK. X1000), MSH2 (mouse, clone G129-1129; BD Pharmingen, San Jose, CA, USA. X500), MSH6 (rabbit, clone EPR3945; GeneTex, Los Angeles, CA, USA. X200), and PMS2 (mouse, clone A16-4; BD Pharmingen. X100). Antigen retrieval was conducted using citrate buffer pH 6.0 for MLH1 and EDTA pH 9.0 for the others (100 °C, 20 min).

Nuclear immunoreactivity for each antibody was evaluated by NH and TM, independently. As there is no standard method for assessing ER-β expression in CRC, the Allred score routinely used in clinical practice for breast cancer was adopted for evaluation. Briefly, nuclear immunoreactivity for ER-β is estimated independently by summing the percentage score, PS, and intensity score, IS, of positively-stained cells (PS: 0%, 0; <1%, 1; <10%, 2; <33%, 3; <67%, 4; ≥67%, 5. IS: weak, 1; medium, 2; strong, 3). For MMR, the loss of the nuclear staining for each antibody (anti-MLH1, anti-MSH2, anti-MSH6, and anti-PMS2) was evaluated. Deficient-MMR (dMMR) was defined as the loss of at least one MMR. Discrepancies were resolved by joint review of the slides.

### 4.3. Examination of ESR2-CA

Deoxyribonucleic acid (DNA) samples were extracted from the pairs of Ca and NonCa frozen tissues from each patient by the phenol/chloroform method. *ESR2*-CA was determined by polymerase chain reaction (PCR) using fluorescein-labeled oligonucleotide primers designed to amplify the polymorphic (CA)n repeat in intron 5 of *ESR2*, as described elsewhere [14]. Briefly, the forward primer was labeled with hexachloro-6-carboxy fluorescein and used together with the tailed reverse primer (5′-FAM-GGT AAA CCA TGG TCT GTA CC-3′, 5′-tail-AAC AAA ATG TTG AAT GAG TGG G-3′). An ABI PRISM 3130 Genetic Analyzer (Applied Biosystems, INC., Foster City, CA, USA) was used for the analyses. Alleles of *ESR2*-CA are presented with the number of CA repeats. Using the same cut-off used in the previous studies, alleles with CA repeats <22 and ≥22 were designated as ‘S’ and ‘L’, respectively, resulting in genotypes SS, LS, and LL [14,15]. To simplify, genotypes ‘SL’ and ‘LL’ were combined as ‘nSS’, and the subjects were finally divided into two genotype categories, ‘SS’ and ‘nSS’. *ESR2*-CA (repeat number or genotype) was compared among each tissue pair (Ca vs. NonCa) or with ER-β expression, considering the background of patients/tumors.

### 4.4. Statistical Methods

The Tukey–Kramer method was used to compare the Allred score (total score, TS) for ER-β between tissue categories classified by tissue type (Ca or NonCa), age/locus category (≥70/right, ≥70Rt; or Non(≥70Rt)), or the MMR status (dMMR or pMMR). Fisher’s exact test using a contingency table was used to compare various nominal variables (*ESR2*-CA genotype or tissue/patient background). In all instances, the statistical software JMP version 12 (SAS Institute, Cary, NC, USA) was used. *p* < 0.05 was considered significant.

## 5. Conclusions

The ≥70Rt cases were characterized by NonCa with a higher rate of the *ESR2*-CA SS genotype or higher ER-β expression compared with Non(≥70Rt) cases. The germline *ESR2*-CA genotype and resulting ER-β expression/estrogen activity seem to affect the type of colon cancer generated or the age of onset/diagnosis of the disease. These may be a clue to resolving the controversy regarding the pathobiological role of estrogen in colorectal cancer.

## Figures and Tables

**Table 1:** Status of mismatch repair protein (MMR) of postmenopausal colon cancer according to patients’ age and tumor locus.

	dMMR n (%)	pMMR n (%)	*p*-Value
**≥70Rt**	**14 (31%)**	**31 (69%)**	0.0005 S *
**Non(≥70Rt)**	**4 (6%)**	**65 (94%)**	
<70Rt	2 (11%)	17 (89%)	
≥70Lt	2 (7%)	25 (93%)	0.0023 S *†
<70Lt	0 (0%)	23 (100%)	

* Fisher’s exact test. † Comparison between four groups (≥70Rt, <70Rt, ≥70Lt, <70Lt). dMMR, deficient-MMR; pMMR, proficient-MMR; ≥70Rt, right-sided case ≥ 70 y/o; <70Rt, right-sided case < 70 y/o; ≥70Lt, left-sided case ≥ 70 y/o; <70Lt, left-sided case < 70 y/o; S, significant (*p* < 0.05). Non(≥70Rt) means <70Rt and ≥70Lt and <70Lt.

**Table 2:** *ESR2*-CA repeat genotype category in non-cancerous (NonCa) or cancerous (Ca) tissue according to the patients’ age/locus and mismatch repair protein (MMR) status of tumors.

	*ESR2*-CA Repeat Genotype Category				
	NonCa		Ca		
	SS n (%)	nSS n (%)	SS n (%)	nSS n (%)	*p*-Value
**≥70Rt** ≥70Rt/dMMR ≥70Rt/pMMR	**22 (49%)** 7 (50%) 15 (48%)	**23 (51%)** 7 (50%) 16 (52%)	**22 (49%)** 7 (50%) 15 (48%)	**23 (51%)** 7 (50%) 16 (52%)	1.0000 * 1.0000 * 1.0000 *
**Non(≥70Rt)** Non(≥70Rt)/dMMR Non(≥70Rt)/pMMR	**12 (17%)** 1 (25%) 11 (17%)	**57 (83%)** 3 (75%) 54 (83%)	**21 (30%)** 3 (75%) 18 (28%)	**48 (70%)** 1 (25%) 47 (72%)	0.1096 * 0.4857 * 0.2058 *
***p*-value ^§^** *p*-value ^§§^	0.0007 S* 0.0026 S*		0.0515 * 0.0484 S*		

* Fisher’s exact test. dMMR, deficient-MMR; pMMR, proficient-MMR; ≥70Rt, right-sided case ≥70 y/o; Non(≥70Rt), other than ≥70Rt; S, significant (*p* < 0.05). ^§^ ≥70Rt vs. Non(≥70Rt), ^§§^ Among ≥ 70Rt/dMMR, ≥70Rt/pMMR, Non(≥70Rt)/dMMR and Non(≥70Rt)/pMMR.

## Data Availability

The data presented in this study are available on request from the corresponding author. The data are not publicly available due to ethical restrictions.
